# Complete Genome Sequences of the Methicillin-Resistant Strain Staphylococcus aureus 17Gst354 and Its Prophage Staphylococcus Phage vB_StaphS-IVBph354

**DOI:** 10.1128/MRA.00586-21

**Published:** 2021-07-08

**Authors:** Sonja Kittl, Isabelle Brodard, Gudrun Overesch, Peter Kuhnert, Joerg Jores, Fabien Labroussaa

**Affiliations:** a Institute of Veterinary Bacteriology, Department of Infectious Diseases and Pathobiology, University of Bern, Bern, Switzerland; University of Rochester School of Medicine and Dentistry

## Abstract

We report the complete 2,783,931-bp circular genome sequence of the human methicillin-resistant strain Staphylococcus aureus 17Gst354, isolated from a nasal swab. The strain possessed an additional 4,397-bp plasmid. Moreover, we induced and sequenced its temperate phage Staphylococcus phage vB_StaphS-IVBph354, which has a circular genome of 41,970 bp.

## ANNOUNCEMENT

The livestock-associated (LA) methicillin-resistant Staphylococcus aureus (MRSA) strain 17Gst354 was isolated from a nasal swab of a healthy Swiss farmer in 2017 (Switzerland) (1). The study was approved by the Ethics Committee for Research of the Canton of Bern (Req-2017-00793). Genomic DNA was extracted as previously reported (2), quantified using a Qubit 4.0 fluorometer (Thermo Fisher Scientific), and then qualified and sized (12- to 15-kb fragments; no shearing) using the Advanced Analytical FEMTO Pulse system (Agilent). Multiplexed SMRTbell libraries were prepared according to the manufacturer’s instructions using the SMRTbell Express template prep kit v2.0. Single-molecule real-time (SMRT) sequencing was performed at the Next Generation Sequencing Platform (University of Bern) on the Sequel system using the Sequel sequencing kit v3.0.

Staphylococcus phage vB_StaphS-IVB354 was purified from strain 17Gst354 through three successive single-plaque isolations after mitomycin C induction, as previously reported (3). Phage genomic DNA was isolated from high-titer lysates using a phenol-chloroform extraction protocol (4). Library preparation was performed using the NEBNext Ultra II directional DNA library prep kit, and Illumina sequencing was performed at Eurofins Genomics (Ebersberg, Germany) on the Illumina NovaSeq 6000 platform in 2 × 150-bp sequencing mode. The raw reads were quality controlled using FastQC v0.11.9 (5) and LongQC v1.2.0 (6) for the Illumina and PacBio reads, respectively. Default parameters were used for all software unless otherwise specified.

A total of 166,155 long reads (average length, 8,827 bp; *N*_50_, 10,217 bp; coverage, 494×) and 3,044,780 paired-end short reads obtained previously (1) (coverage, 297×; SRA accession number SRX6491214) were used to assemble the complete 2,783,931-bp chromosome (G+C content, 32.9%) of strain 17Gst354 using Unicycler v0.4.4 (7). The chromosome was rotated to the first nucleotide of the start codon of the *dnaA* gene using the fixstart command of Circlator v1.5.5 (8). The chromosome sequence was annotated using the NCBI Prokaryotic Genome Annotation Pipeline (PGAP) (9). A total of 2,754 open reading frames (ORFs) were detected and included the genes *mecA* [staphylococcal cassette chromosome *mec* (SCC*mec*) type IVa(2B), as determined using SCCmecFinder v1.2 (https://cge.cbs.dtu.dk/services/SCCmecFinder/)], and *blaZ* (beta-lactamase), as well as 59 tRNAs and a set of six 5S, five 16S, and five 23S rRNAs. A 4,397-bp plasmid was also present. A BLASTN analysis (10) showed 99% identity with the plasmid pRIVM4390 previously isolated from other MRSA strains (11).

The presence of two prophages was detected *in silico* using PHASTER (12) at positions 337407 to 383619 and 2001104 to 2046421. The latter sequence matched to the genome sequence of Staphylococcus phage vB_StaphS-IVBph354. This genome was assembled into one circular contig from a total of 4,789,426 paired-end short reads using Unicycler v0.4.4. The mean depth (coverage, 34,061×) was determined using the coverage command of SAMtools v1.11 (13) after mapping with the Burrows-Wheeler Alignment (BWA) tool v0.7.17 (14).

Phage vB_StaphS-IVBph354 has a genome of 41,970 bp with a G+C content of 33% and includes 65 ORFs. Whole-genome alignment using EMBOSS Stretcher (15) showed that the vB_StaphS-IVBph354 genome is identical to that of another β-hemolysin-converting integrase group 3 (ΦSa3) phage, Staphylococcus phage P282 (GenBank accession number NC_048634.1) (16), except that the vB_StaphS-IVBph354 genome carries a classical *attB* site (5′-TGTATCCAAACTGG-3′) and a frameshift insertion (5′-GAGCGAAAGA-3′), which extend the corresponding ORF (locus tag, HWA89_gp54).

### Data availability.

The Illumina reads are available under the following SRA accession numbers: SRX6491214 (strain 17Gst354) and SRX10584153 (Staphylococcus phage vB_StaphS-IVBph354). The PacBio reads are available under SRX10576957. The assemblies can be accessed under the GenBank accession numbers MW924889 (Staphylococcus phage vB_StaphS-IVBph354), CP073065 (strain 17Gst354), and CP073064 (17Gst354 plasmid).
